# Normal-pressure hydrocephalus: A critical review

**DOI:** 10.1590/1980-57642018dn13-020001

**Published:** 2019

**Authors:** Louise Makarem Oliveira, Ricardo Nitrini, Gustavo C. Román

**Affiliations:** 1 Medical Student, School of Medicine, Federal University of Amazonas (UFAM), Manaus, AM, Brazil.; 2 Professor of Neurology, Department of Neurology, University of São Paulo Medical School, São Paulo, SP, Brazil.; 3 The Jack S. Blanton Distinguished Endowed Chair, Neurological Institute Houston, Methodist Hospital, Professor of Neurology Weill Cornell Medical College Methodist Neurological Institute, USA.

**Keywords:** cerebral blood flow, falls, incontinence, normal-pressure hydrocephalus, reversible dementia, spinal tap test, fluxo sanguíneo cerebral, quedas, incontinência, hidrocefalia de pressão normal, demência reversível, teste de punção lombar *(tap test)*

## Abstract

Normal-pressure hydrocephalus (NPH) is a potentially reversible syndrome characterized by enlarged cerebral ventricles (ventriculomegaly), cognitive impairment, gait apraxia and urinary incontinence. A critical review of the concept, pathophysiology, diagnosis, and treatment of both idiopathic and secondary NPH was conducted. We searched Medline and PubMed databases from January 2012 to December 2018 using the keywords “normal-pressure hydrocephalus” / “idiopathic normal-pressure hydrocephalus” / “secondary normal-pressure hydrocephalus” / “NPH” / “ventriculoperitoneal shunt”. The initial search produced 341 hits. After careful selection, a total of 54 articles were chosen and additional relevant studies were included during the process of writing this article. NPH is an important cause of potentially reversible dementia, frequent falls and recurrent urinary infections in the elderly. The clinical and imaging features of NPH may be incomplete or nonspecific, posing a diagnostic challenge for medical doctors and often requiring expert assessment to minimize unsuccessful surgical treatments. Recent advances resulting from the use of non-invasive MRI methods for quantifying cerebral blood flow, in particular arterial spin-labeling (ASL), and the frequent association of NPH and obstructive sleep apnea (OSA), offer new avenues to understand and treat NPH.

## RESUMO.

A hidrocefalia de pressão normal (HPN) é uma síndrome potencialmente reversível marcada por ventrículos cerebrais alargados (ventriculomegalia), declínio cognitivo, apraxia da marcha e incontinência urinária. Revisar criticamente o conceito, a fisiopatologia, o diagnóstico e o tratamento da HPN idiopática e secundária. Os autores acessaram as bases de dados Medline e Pubmed entre janeiro de 2012 e dezembro de 2018, utilizando as palavras-chave *“normal-pressure hydrocephalus” / “idiopathic normal-pressure hydrocephalus” / “secondary normal-pressure hydrocephalus”* / “NPH” / *“ventriculoperitoneal shunt”*. A busca inicial resultou em 341 artigos. Após cuidadosa seleção, 54 estudos foram escolhidos e pesquisas adicionais foram incluídas durante o processo de elaboração do manuscrito. A HPN é uma importante causa de demência potencialmente reversível, quedas frequentes e infecção urinária recorrente em idosos. As características clínicas e de imagem da HPN podem ser incompletas ou inespecíficas, de modo que este se torna um diagnóstico difícil para médicos. Não raro uma avaliação por especialista é necessária, visando minimizar tratamentos cirúrgicos ineficazes. Avanços recentes advindos do uso não invasivo de ressonância magnética para quantificação do fluxo sanguíneo cerebral, em particular *arterial spin-labeling* (ASL), assim como a usual associação entre HPN e apneia obstrutiva do sono representam novos meios de entender e de tratar a HPN.

In 1761, Giambattista Morgagni described 3 autopsy cases of “chronic senile hydrocephalus” in subjects older than 60 years of age.^1^ However, the clinical syndrome of normal-pressure hydrocephalus (NPH) was only recognized 53 years ago by Hakim et al.^2^^,^ 3

The names Hakim syndrome, Hakim triad and Hakim-Adams syndrome are often used to acknowledge the discovery by the Colombian neurosurgeon, Salomón Hakim, who published his results together with the American neurologist, Raymond D. Adams.^4^

## DEFINITION

NPH is a potentially reversible syndrome characterized clinically by enlarged cerebral ventricles (ventriculomegaly), cognitive impairment, gait apraxia and urinary incontinence.^2^^,^ 3 In 1975, a decade after the initial NPH publications, Shenkin et al.^5^ reported symptomatic hydrocephalus in adults without increased intracranial pressure (i.e., “normal pressure”) occurring in the absence of other obvious causes. For the first time, they classified these cases as *idiopathic* NPH (iNPH) and reported that in elderly patients (average age 68 years, range 52-83) with iNPH manifested by cognitive symptoms of “senile dementia,” 64% (18/28) improved after cerebrospinal fluid (CSF) shunting.

The usual NPH classification into *idiopathic* (iNPH), accounting for about 50% of cases, and *secondary* (sNPH), resulting from subarachnoid hemorrhage, meningitis, intracerebral hemorrhage, brain tumor or head trauma,^3^^,^ 6 ^,^ 7 is not helpful from a practical viewpoint, mainly because the actual pathogenesis of the NPH syndrome remains unclear. Whereas iNPH is primarily observed in adults older than 60 years, sNPH can occur at any age.^8^^,^ 9 In both cases, however, men and women are equally affected.^9^ Other than ventriculomegaly, there is no definitive pathological or radiological diagnostic finding for NPH, which is frequently over-suspected and under-confirmed, based only on positive response to CSF shunting.

The diagnostic criteria for NPH remain a topic of discussion. The numerous controversies surrounding this disease led to an interesting dichotomy: while some consider the disorder as the most common type of hydrocephalus in adults,^9^ others, especially in recent years, have been advocating against its existence.^10^^-^ 13 In order to understand this duality, it is not only necessary to revisit the first description of the condition, but also to thoroughly assess recent data.

The aim of this review was to evaluate current knowledge on the two forms of NPH, their etiology, diagnosis, pathophysiology and treatment, highlighting novel findings within the last 5 years; this process should ultimately help to answer the question on the merit of the assumption that NPH is a frequent cause of dementia, abnormal gait and falls in the elderly.

## METHODS

We searched Medline and PubMed from January 2012 to December 2018 for relevant articles using the keywords “normal-pressure hydrocephalus,” or “idiopathic normal-pressure hydrocephalus,” or “secondary normal-pressure hydrocephalus,” or “NPH,” or “ventriculoperitoneal shunt (VPS).” The initial search produced 341 hits and, after selection based on the abstracts, a total of 54 articles on etiology, diagnosis, pathophysiology or treatment were chosen and reviewed. Additional references were obtained from these articles and from the authors of this review.

## IDIOPATHIC NORMAL-PRESSURE HYDROCEPHALUS (iNPH)

### Concept, pathophysiology, and diagnosis

Since its description in 1965, the concept of iNPH has been a recurrent focus of discussion. Of the 6 original patients, 2 were post-traumatic hydrocephalus (males, ages 16 and 43 years), 1 woman (age 63) had suspected meningeal carcinomatosis, 1 man (age 62) with a III^rd^ ventricle cyst, and only 2 were idiopathic NPH (male, age 52 and woman, age 63). In addition to symptoms of brain edema and traumatic brain injury, including akinetic mutism, they all presented with frontal dementia plus gait disability resembling Bruns apraxia or frontal ataxia, and frontal-type urinary and fecal incontinence. All of these symptoms responded dramatically to neurosurgical treatment with ventriculoatrial shunting^14^ or with the Torkildsen procedure (ventriculocisternostomy). All patients had normal opening CSF pressure upon spinal tap and most improved after the draining of CSF. The common finding in all the original cases, demonstrated by pneumoencephalograms, was a symmetrical and massive enlargement of the entire ventricular system - including the aqueduct and IV^th^ ventricle - without air in the subarachnoid space. This finding suggested communicating hydrocephalus, hence the name “normal-pressure” hydrocephalus used for the first time.^10^^,^ 14

As mentioned above, a critical evaluation of these initial descriptions reveals that the mechanism of CSF flow obstruction that caused the hydrocephalus was known in all but 2 of the reported cases.^10^^,^ 14 Additionally, 3 patients had a recorded CSF opening pressure of 180 mmH_2_O, a value that, although normal, is very close to the upper acceptable limit. It is also possible that 180 mmH_2_O was not the peak intracranial pressure due to the CSF dynamics and possibility of partial obstruction.^10^ Hence, Hakim and Adams and collaborators,^10^^,^ 14 described a mixture of what would now be called sNPH and iNPH cases; thus, from a historical viewpoint, the current separation of NPH into these 2 forms might not be justified, as explained later.

Over the years, the term iNPH has been almost indiscriminately used for all individuals who present with “unexplained” ventriculomegaly detected by brain imaging including computed tomography (CT) or magnetic resonance imaging (MRI), associated with the classic triad comprising cognitive impairment, gait disturbance, and incontinence.^2^^,^ 10 ^-^ 14 In reality, the number of “unexplained” cases might vary according to the intensity of the etiological search, including the use of CSF biomarkers and even brain biopsy.

According to International Guidelines the following key imaging features should be employed for the diagnosis of NPH: 15 ^,^ 16

1. Ventricular enlargement with Evans’s index >0.3 (Figure 1A).
  **Figure 1:**
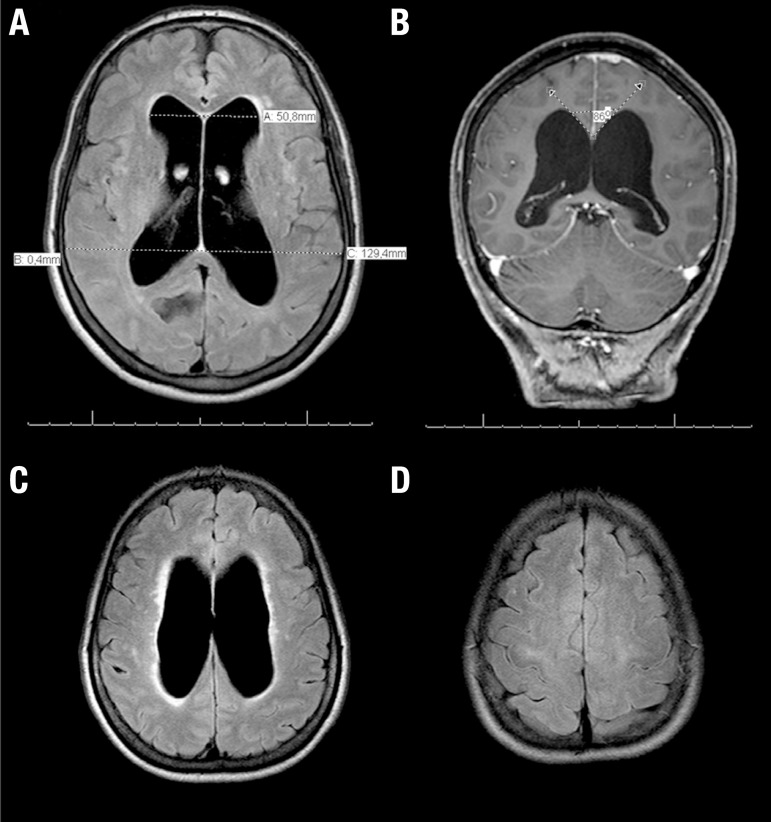
Neuroimaging in NPH **(A)** Axial FLAIR MRI scan showing a significant ventriculomegaly with increased Evans Index, the ratio of maximum width of the frontal horns of the lateral ventricles and maximal internal diameter of skull at the same level on axial CT or MRI images. In this case, Evans index is 0.39 (abnormal > 0.3) **(B)** T1-weighted coronal gadolinium-enhanced MRI scan showing reduced callosal angle. **(C)** Axial FLAIR MRI scan revealing enlarged lateral ventricles with bright signal in the surrounding white matter, suggestive of transependymal edema. **(D)** Axial FLAIR MRI showing narrowing of the sulci and subarachnoid spaces over the high convexity and midline surface in the frontoparietal regions.
2. Absence of macroscopic obstruction to CSF flow.
3. At least one of these supporting features:

- Enlarged temporal horns of the lateral ventricles not entirely due to hippocampus atrophy;
- Callosal angle of 40° or greater (Figure 1B);
- Periventricular signal changes on CT and MRI due to altered brain water content and not entirely attributable to microvascular ischemic changes or demyelination (Figure 1C);
- Flow void in the Sylvian aqueduct or fourth ventricle on MRI.

The Japanese Guidelines for the diagnosis of NPH did not regard periventricular changes as relevant for the diagnosis, but included two other key imaging features: narrowing of the sulci and subarachnoid spaces over the high convexity and midline surface of the brain (Figure 1D); and enlarged Sylvian fissures and basal cisterns.^17^

In 2017, Andersson et al.^18^ evaluated the aforementioned guidelines and found remarkable discrepancies between these and the neurologists. Overall, this paper stated that the Japanese guidelines were more concordant with the professionals, which can be partially explained by the comparative lack of specificity that marks the International Guidelines - neither radiological nor clinical criteria are particular enough, with only one symptom of the classical triad being sufficient for “possible” disease, whereas at least two are necessary according to the Japanese guidelines. Furthermore, this study emphasized the need to review the current guidelines in order to produce a common, more objective, diagnostic system.^18^

### Differential diagnosis of NPH

When ventriculomegaly is excluded, the NPH symptomatology becomes nonspecific. After all, dementia resulting from various causes occurs in about 35% of people aged over 70 years;^19^ urinary incontinence is present in almost 40% of women and 20% of men aged over 60 years;^20^ and, gait impairment is observed in almost 20% of those aged over 75 years.^21^ Hence, it is important to include in the differential diagnosis conditions such as Alzheimer’s disease (AD), atypical Parkinsonism, dementia with Lewy bodies (DLB), progressive supranuclear palsy (PSP), and vascular dementia (VaD). These, and other entities, should be adequately excluded in order to avoid incorrect treatment.^22^^-^ 24

The clinical differentiation between iNPH and the aforementioned conditions may be very subtle, requiring specialized assessment by dementia experts.

### Alzheimer’s disease

The clinical presentation of normal pressure hydrocephalus is linked to impaired periventricular blood flow, as well as to interstitial edema, microvascular infarctions, neuronal injury, and gliosis,^25^ as will be discussed further in the pathophysiology section. The neuronal degeneration is probably due to enlarged ventricles, impaired blood-brain barrier, and reduced CSF clearance, which leads to accumulation of neurotoxins such as b-amyloid and tau-protein.^26^^,^ 27 This jeopardized turnover can explain the Alzheimer-like changes in the cortex of iNPH patients and of rats with chronic hydrocephalus, as observed by Del Bigio et al.^28^ and Klinge et al.^29^

Despite being important conditions in the differential diagnosis, AD and the NPH syndrome can differ greatly in terms of clinical presentation. In 2016, Damasceno^16^ reinforced these differences - highlighting that, whilst in AD a “subcortical” type of cognitive impairment predominates, classically characterized by a dysexecutive syndrome, associated with inattention, apathy, memory impairment, and psychomotor slowing, NPH is marked by the presence of “cortical” signs - such as hippocampal amnesia, agnosia, apraxia, and aphasia. In addition, AD dementia often precedes and overshadows motor and urinary symptoms.^30^

Brain biopsies performed on patients treated successfully with CSF derivation for iNPH, have shown various underlying brain pathologies, mainly AD, indicating that the 2 conditions are not mutually exclusive. In fact, about 19%^31^^,^ 32 - and as much as 24%^33^- of iNPH patients undergoing CSF shunt insertion have neuropathologically-confirmed diagnosis of AD.^34^ Interestingly, the biopsy-positive diagnosis does not initially affect the beneficial effect of the shunt on the iNPH symptoms.^34^

However, 32% of patients with moderate-to-severe AD pathology had worse baseline cognitive test scores and less postoperative improvement of NPH symptoms 4 months after VPS.^35^ Because of co-occurrence of NPH and AD, the symptoms of the NPH triad may be observed in late stages of AD. Conversely, about 75% of severely demented NPH patients experience overlapping AD characteristics.^22^^-^ 24 Espay et al.^12^ suggested that, in patients older than 65 years diagnosed with iNPH, both the symptoms and the brain atrophy could be explained by an underlying neurodegenerative condition rather than by NPH.^12^ However, as summarized by Román^36^ in 2016, although AD and other neurodegenerative pathologies do occur in elderly patients with confirmed iNPH, suspected AD should not automatically exclude the patient from treatment of NPH. In fact, the progression of neurodegeneration in AD does not explain the occurrence of abnormal gait and incontinence, given that the tau deposits, neurofibrillary tangles, neuronal disconnection, and cortical atrophy, typically follow a trans-synaptic progression from early lesions in the entorhinal cortex to hippocampus-parahippocampal cortex and limbic system, finally affecting neocortical areas. Even at Braak & Braak stages V-VI of advanced AD there is minimal involvement of supplementary motor cortical areas that could produce alterations of gait or of bladder control.

The treatment of NPH can be highly beneficial, both from the viewpoint of prevention of frequent falls, head injury, subdural hematomas, trauma and hip fractures in the elderly, as well as for the improvement of quality of life with disappearance of incontinence, control of the risk of repeated urinary tract infections and sepsis, freedom of ambulation, and in some selected cases, cognitive improvement.^36^

Despite the existence of numerous diagnostic criteria for NPH,^37^^-^ 40 including Hashimoto’s MRI-based criteria for NPH,^34^^,^ 40 there is lack of universal agreement on the required elements for diagnosis.

### Pathophysiology of iNPH

In the original descriptions, Hakim et al.^41^ emphasized that ventriculomegaly is the central element in the clinical syndrome due to the hydraulic pressure effect. The explanation is based on Pascal’s law of hydrodynamics, whereby the *force* exerted by the CSF on the walls of the ventricles is equal to the product of the *pressure* of the fluid and the *area* of the wall: F = P × A. In the original example, they wrote that the CSF opening pressure is transmitted to every square centimeter of the surface of the container; therefore, a pressure of 150 mmH_2_O exerts a force of 300 mmH_2_O on the surrounding brain when the ventricles’ surface area is 120 cm^2^ compared with half that value (150 mmH_2_O) with the same opening pressure on ventricles with a normal surface of 60 cm^2^. Hakim, Venegas & Burton^41^ (1976) concluded: “It is important to recognize that some aspects of intracranial physiopathology can be explained through classical concepts of physics, prior to attempting to interpret such processes solely in terms of biological or auto-regulatory phenomena.”^41^

The force exerted on the ventricles is transmitted centrifugally, compressing the brain and elevating the *transmantle pressure,* i.e., the difference between ventricular pressure and the pressure over the cerebral convexity. The result is a global decrease in cerebral perfusion from the centrifugal transmantle pressure, given that most of the arterial cerebral blood flow (CBF) is centripetal, i.e. from the subarachnoid space towards the center of the brain.^42^^,^ 43

Owler and Pickard^44^ reviewed studies of cerebral perfusion in hydrocephalus and concluded that multiple methods have shown a consistent decrease in CBF, mainly in the frontal cortex, periventricular white matter, basal ganglia, and thalami.^45^^-^ 51

Enhancement of cerebral perfusion in periventricular white matter and basal ganglia has been described after CSF removal^52^ or following surgical CSF shunt treatment.^53^^-^ 55

Arterial Spin-Labeling (ASL) perfusion imaging is a novel non-invasive MRI technique that requires neither contrast media nor isotopes to measure CBF. ASL-MRI generates an endogenous contrast by using radiofrequency pulses that “label” water proton spins in blood circulating in carotid and vertebral arteries at the base of the skull. CBF-ASL-MRI images are obtained by subtracting labeled and unlabeled spin exchanges in the brain tissue yielding a map of regional CBF (rCBF) quantified in mL/100g/min. We use 3D pseudo-continuous ASL (pCASL) with spiral acquisition before and after the tap test for NPH. Also, CBF-ASL-MRI has been used to demonstrate regional decrease of CBF in NPH^56^^,^ 57 and in hydrocephalus secondary to posterior fossa tumors in children,^58^ as well as to determine the beneficial effects of acetazolamide treatment in NPH.^59^

**Figure 2:**
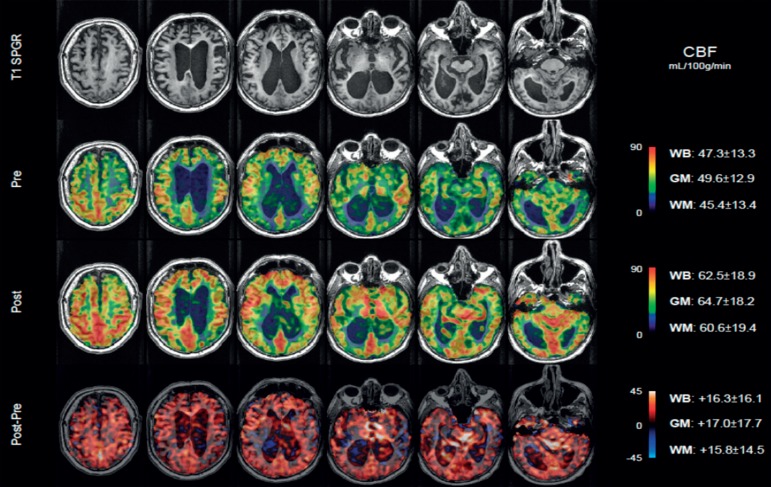
An example of ASL-MRI illustrating a positive correlation between enhanced CBF and clinical improvement after large-volume spinal tap (unpublished data).

Using ASL-MRI, we have demonstrated a positive correlation between enhanced CBF (Figure 2) and clinical improvement after large-volume spinal tap (unpublished data).

It should be noted that the CSF is called “the third circulation” because of the constant interaction between cerebral arterial circulation, CSF circulation, and venous circulation. The concept of intracranial venous hypertension leading to decreased CSF absorption and hydrocephalus is critical to fully understand the pathogenesis of NPH.^60^

Venous hypertension in intracranial circulation hinders CSF absorption through the arachnoid villae in the dural sinuses.^60^^-^ 64

Also, venous hypertension alters intracranial compliance and changes CSF dynamics, affecting the intracranial Windkessel effect from brain viscoelastic properties.^45^^,^ 60 ^,^ 61 ^,^ 65

Other than the effects of ventriculomegaly, in 2006, Malm and Eklund,^66^ described some of the physiological processes potentially involved in iNPH, including reversible dysfunction of neuronal and glial mechanisms; they pointed to increased intracranial pressure pulsatility and CSF outflow resistance as probable triggers.^66^

They also observed that the clinical features of iNPH most likely result from periventricular, frontal or subcortical impairment.^66^ These areas are mainly supplied by periventricular end arteries, which are easily affected by ischemia - hence the clinical symptoms and hyperintensities seen on patient MRIs.^9^^,^ 66 Infarctions, however, are less frequent, justifying the reversibility after shunt procedures.^9^^,^ 66

In 2010, Ott et al.^67^ correlated the abnormal dilatation of the ventricles with limited re-absorption of cerebrospinal fluid - the subsequent stasis being responsible for defective metabolic clearance.^23^^,^ 67 Increased aqueductal CSF flow is considered a positive finding in patients with NPH.^68^

Recently, an association between NPH and the glymphatic system has emerged, attempting to link reduced intracranial compliance and diminished arterial pulsations with inefficient glymphatic flow.^39^^,^ 69 ^,^ 70 If confirmed, this could partially explain the frequent occurrence of dementia as a prominent characteristic of this disorder, as well as the higher incidence of AD in NPH patients.^39^^,^ 71 Nevertheless, iNPH pathogenesis remains unclear. Very recently, Román et al.^72^ found a strong correlation between obstructive sleep apnea and NPH.

The mechanisms induced by OSA cause almost total absence of REM and delta sleep, affecting glymphatic flow; also, sleep disordered breathing produces cerebral venous hypertension due to increases in central venous pressure. The postulated net result is a decrease in CSF outflow, leading to hydrocephalus. Nocturnal polysomnogram is therefore indicated in the evaluation of patients with suspected NPH.

### Predictive tests and treatment

NPH is classically defined as a communicating form of hydrocephalus^37^^,^ 73 ^-^ 75 without a fully effective non-invasive treatment.^22^^,^ 76 ^-^ 80 However, because of the new hydrodynamic concept of hydrocephalus,^81^^,^ 82 as opposed to the classical dichotomy proposed by Dandy, 82 ^,^ 83 the use of endoscopic third ventriculostomy (ETV), which is the golden standard for non-communicating cases,^84^ has been reported in selected cases of NPH, as an attempt to restore normal intracranial compliance and pulsatility, normalizing the CSF dynamics.^81^^,^ 82 ^,^ 85 ^-^ 88 The results showed an effectiveness rate of ETV ranging from 21% to 72%.^81^^,^ 82 ^,^ 85 ^-^ 88 Nevertheless, the available data is insufficient to determine whether or not this surgery is superior to VPS, the established treatment for NPH.

CSF shunting and subsequent drainage continues to be the first-line therapy for NPH, with the symptoms usually improving after the intervention. Many authors consider the responsiveness to shunting as the main difference between iNPH and sNPH, where clinical improvement is seen in about 50% of individuals with iNPH and in up to 70% of those with sNPH.^77^

### Large-volume lumbar puncture (Tap Test)

A number of physiologically based tests have been developed^9^ to identify CSF flow abnormalities and those patients most likely to respond to CSF shunts. The tap test or large-volume lumbar puncture (LVLP) is one of the most disseminated worldwide, for it is easily performed and cost-effective.

Adams et al.^3^ were the first to describe the improvement of fleeting symptoms in NPH patients who underwent lumbar puncture. Refinement of the technique, however, occurred years later, with Wikkelsø et al.^89^ responsible for adding quantitative methods to the procedure in order to evaluate cognition and gait.

The tap test works by temporarily decreasing intraventricular pressure, mimicking the effect of a shunting procedure, allowing the physician to evaluate the patient’s response to a substantial (50 ml) CSF removal. Table 1 summarizes the procedure as performed at the Neurological Institute of Houston Methodist Hospital.^36^

**Table 1:** Houston methodist hospital protocol for patients with suspected NPH*.

Pre-lumbar puncture	
• Cognitive evaluation by neuropsychologist	
• Physical therapy evaluation: gait & balance	
• Sphincter continence	
MRI brain, non-contrast, with ASL for CBF	
Large-volume LP: 50 mL under fluoroscopy	
Post-lumbar puncture	
• Repeat pre-LP protocol within 24 hours	
• Caregiver global impression of change	

* Román NPH Protocol.

Given the diagnostic uncertainties mentioned earlier, there are major advantages in securing consensus recommendations from a team that includes specialists in neurology, neuropsychology, physical therapy, neuroradiology and neurosurgery. Moreover, using quantifiable measurements (balance and gait, cognitive test scores, episodes of incontinence, CBF in mL/100g/min), allows objective judgment of each of the test components. The Román Protocol for the LVLP diagnostic test for NPH is performed as follows:^36^

*Neuropsychology.* On the day prior to the LVLP, a clinical neuropsychologist evaluates the following cognitive domains: global cognition, memory, orientation, language, praxis, and executive function. Twenty-four hours after the LVLP, the same specialist performs the second evaluation, modified to avoid learning and practice effects.
*Physical Therapy.* On the day of admission for the LVLP, a pre-trained Physical Therapist examines the patient gait and balance prior to the LVLP using the scores from the Tinetti test^90^ and the Berg Balance scale (BBS).^91^
*Sphincter Control.* During the period of in-hospital observation (24 hours), pre- and post-LVLP, the patient´s accompanying relative is instructed to notify the nurse if the subject asks to void or to evacuate, or if incontinence occurred. The number of such events in the 24 hours pre- and post-LVLP is recorded.
*Non-enhanced brain MRI with CBF-ASL:* The baseline non-enhanced brain MRI test is performed on the days leading up to the LVLP and is repeated within 24 hours after the tap test. It is usually well tolerated; does not expose the patient to X-ray radiation, requires no intravenous contrast medium, and can be repeated as often as needed. The only limitation is that MRI cannot be performed in patients with cardiac pacemakers or defibrillators.
*Large-Volume Lumbar Puncture.* A neuroradiologist performs a routine lumbar puncture under fluoroscopy with an 18- or 20-gauge spinal needle;^6^ ideally, a total of 50 mL of CSF is collected. Opening and closing pressures are recorded and CSF laboratory examinations are obtained, including levels of b-Amyloid and Tau protein.

The final diagnosis and therapeutic decision should be the professional responsibility of the trained neurologist or neurosurgeon in charge of the patient after considering the results of each component of the tap test. Usually, surgical treatment with insertion of a ventriculoperitoneal shunt (VPS) is recommended only for patients that present clear improvement in gait post LVLP, usually with concurrent improvement in bladder control. Few patients show improvement in cognitive evaluation within the 24 hours post LVLP. For patients considered to be non-surgical candidates, and for those that decline surgery, the use of acetazolamide (Diamox®) is recommended at relatively low doses (125-500 mg/day).^92^

Despite its novel nature and considerable advantages, the Román Protocol can be associated with some drawbacks - predominantly related to cost-effectiveness - such as requiring a multidisciplinary team, multiple procedures, and a 24-hour stay in a hospital.

Although associated with a short learning curve, the tap test should be performed by trained professionals.

Professionals must be aware that a lack of response on this test does not contraindicate the surgical procedure,^9^^,^ 93 as was underpinned by Wikkelsø et al.^94^ This multicenter European study, which concluded that the tap test is valid for selecting patients for surgery, but not for excluding them from the treatment, was based on a combined CSF dynamic test that included the results of a 50 ml CSF tap test analysis. In this test, patient gait was assessed three hours after CSF drainage, by measuring the number of steps and seconds needed to walk 10m at free speed.

Physicians should also bear in mind that repeating the tap test at a later date is possible. Repeated large-volume lumbar puncture is an alternative treatment seldom used.

External lumbar drainage and infusion testing are other predictive tests that tend to require more expertise. These tests are more frequently used in European countries. An association between augmentation of CBF following glycerol administration and a favorable response to shunt procedures has been proposed.^95^ Intracranial pressure (ICP) monitoring is an invasive neurosurgical method used as a diagnostic and predictive test for iNPH; waveform alterations and unstable ICP correlate with a 50-90% response to VPS.^39^^,^ 96 ^-^ 99

Adequate selection of surgical candidates leads to a 90% chance of success, according to a recent review.^9^ Even so, many studies fail to mention which predictive tests were used in patient assessment. Moreover, CSF pressure figures are commonly unreported,^10^ and over 40% of patients with iNPH do not have all the components of the traditional syndrome. Some studies require just one or two out of the triad of components to recommend VPS.^100^

In light of this, and considering that surgery may be an effective placebo, recent suggestions have been made to conduct randomized, double-blind, placebo-controlled clinical trials comparing the efficacy of shunting and placebo procedures for iNPH,^10^^,^ 12 in a bid to provide solid evidence-based practice recommendations.

Also, shunting procedures are not free of complications and can be associated with significant adverse effects (AEs), including subdural hematomas and hygromas, shunt and CNS infection, complex partial seizures, over-drainage, and prolonged post-operative delirium.^101^^,^ 102 Less common consequences include death and delayed postoperative pneumocephalus.^101^^,^ 103

### Recent Brazilian experience with NPH and Tap-test

In 2018, Souza et al.^104^ investigated the impact of the CSF tap test on the gait of patients diagnosed with iNPH. The tap test performed involved the removal of CSF for two consecutive days, with a 24-hour interval between the lumbar punctures. Each procedure removed 30 mL of cerebrospinal fluid. The patient’s gait was assessed at two timepoints: prior to the first LP, and three hours after the second procedure. The whole test lasted for about 48 hours. This study revealed that gait speed was the most responsive parameter to the test.

Souza et al.’s study was critically reviewed by Damasceno^105^ in an editorial, where the aforementioned result was found to be in accordance was the available literature. The need to determine whether other postural or gait parameters could better predict response to surgery was reinforced. Additionally, Damasceno also supported repeated or continuous three-day external lumbar drainage (minimum of 150 ml CSF drained daily) as a way to enhance tap test sensitivity (50-100%) while maintaining a high positive predictive value (80-100%), compared to the one-tap CSF tap test with low sensitivity (26-61%).^15^^,^ 105

### Secondary normal pressure hydrocephalus

Secondary normal pressure hydrocephalus encompasses all cases in which an etiology is identified.^11^ It has yet to be determined how long after the inciting event the symptoms must appear in order to establish a cause-effect relationship, with opinions varying from immediate to delayed onsets.^106^^,^ 107 Engel et al.^108^ found that an elevated Evans’ ratio^109^ was the most common radiological finding preceding the onset of symptoms.

Along the same lines, a population-based MRI study^110^ of Japanese elderly ≥61 years of age found a prevalence of 6.46% of enlarged ventricles measuring >0.3 on the Evans’ index; although only 0.51% had iNPH symptoms, after 4-8 years 25% of the asymptomatic subjects developed symptoms consistent with iNPH. In agreement with the above, Jaraj et al.^111^ conducted a study in Sweden of 1,238 European elderly subjects (≥70 years) diagnosed with iNPH according to American-European guidelines^37^ (i.e., ventriculomegaly, gait disturbance and either Mini-Mental State Examination (MMSE)^112^ ≤25 or urinary incontinence). The study found an Evans’ Index >0.3 in 256 (20.7%) persons of the group. The prevalence of probable NPH was 200/100,000 in those aged 70-79 years and 5,900/100,000 in those aged 80 years and older, with no gender difference.^111^

Thus, based on firm epidemiological data, it can be concluded that iNPH is a frequent disorder in the elderly, with an average age of onset of about 70 years; more importantly, between 60% and 80% of patients improve with shunt surgery, but only a minority receive the benefit of the surgical treatment with CSF shunt.^9^^,^ 113

Because sNPH may result from several different causes, it has become difficult to create practical guidelines for optimizing the treatment and diagnosis of the disease.^11^ In both forms of NPH, the diagnosis remains based on clinical history, neurological examination, and brain imaging, while the treatment is mainly CSF shunt - involving procedures such as ventriculoperitoneal and ventriculoatrial shunts.

A recent review by Daou et al.^11^ assessed 64 studies and showed that subarachnoid hemorrhage (SAH) was the leading cause of sNPH (46.5%), followed by head trauma (29%), intracranial malignancies - and resection surgeries - (6.2%). Intracerebral hemorrhage, Paget’s disease, cerebrovascular diseases, aqueductal stenosis, and radiosurgery were responsible for the other cases.

Up to 37% of the patients with SAH developed chronic hydrocephalus,^107^ and the basal cisterns and arachnoid villi fibrosis may determine NPH development.^114^ Posttraumatic hydrocephalus comprises a varied group of injuries that ultimately impairs CSF flow. On the other hand, brain tumors and inflammatory processes, including neurocysticercosis in tropical countries, increase CSF viscosity due to proteins and other products^115^ - hence, CSF reabsorption by arachnoid granulations is jeopardized, leading to NPH.

Because some studies have evaluated iNPH and sNPH as the same condition, it has also been questioned whether or not the sNPH concept is even valid, and to what extent it is useful for differentiating sNPH from iNPH. We basically agree with this concept, given that ventriculomegaly is the central axis of the NPH syndrome. However, Daou et al. concluded that, despite differences in outcome, iNPH and sNPH should not be treated as completely separate entities.^11^

Taking everything into account, the diagnosis of NPH should always be considered when facing a suggestive clinical presentation. This often occurs in the emergency room when routine brain CT in an elderly patient undergoing evaluation for trauma surprisingly discloses ventriculomegaly, or in the evaluation of patients with gait disorders or cognitive decline in outpatient clinics. It is recommended to always search for an etiology and, even when one fails to be found, the diagnosis of NPH should be properly - and rapidly - addressed.

## CONCLUSION

Based on a comprehensive review of the recent literature, we conclude that the clinical syndrome of NPH is the same in both iNPH and sNPH. The separation into idiopathic and secondary NPH has resulted in a difference of approach to the syndrome, manifested by a sense of urgent evaluation and treatment only for sNPH. The initial step in the diagnostic process is the evaluation of ventriculomegaly on brain imaging.

Both the concept and the management of normal-pressure hydrocephalus remain a topic of discussion, marked by several controversies. Nonetheless, it is clear from population-based epidemiological studies that iNPH is more common than previously thought. Therefore, each situation must be thoroughly and individually assessed in order to prevent misdiagnosis and incorrect treatment. The confirmation of this disease is rather complex, requiring the involvement of dementia experts, because iNPH has no particular imaging, clinical or pathological features, and there are no definitive tests capable of accurately diagnosing the condition. It is also essential to keep in mind that to minimize errors the tap test should be performed by trained professionals. Overall, secondary NPH is associated with better outcomes, which is partially explained by swift intervention with adequate VPS placement, which remains the first-line of treatment. NPH may still be considered a potentially reversible cause of dementia; however, a randomized, placebo-controlled trial of shunting procedures should be conducted to finally prove - or refute - the true efficacy of surgical interventions.
