# Errata

**DOI:** 10.1590/1980-57642018dn13-030016

**Published:** 2019

## The authors noticed an inaccuracy in the paper entitled “Normal-pressure hydrocephalus: a critical review” by Oliveira et al.

In the “Alzheimer's disease” section, when you read:

(...) whilst in **AD** a “subcortical” type of cognitive impairment predominates, classically characterized by a dysexecutive syndrome, associated with inattention, apathy, memory impairment, and psychomotor slowing, **NPH** is marked by the presence of “cortical” signs - such as hippocampal amnesia, agnosia, apraxia, and aphasia.

Read:

(...) whilst in **NPH** a “subcortical” type of cognitive impairment predominates, classically characterized by a dysexecutive syndrome, associated with inattention, apathy, memory impairment, and psychomotor slowing, **AD** is marked by the presence of “cortical” signs - such as hippocampal amnesia, agnosia, apraxia, and aphasia.

Louise Makarem Oliveira, Ricardo Nitrini, Gustavo C. Roman. Normal-pressure hydrocephalus: a critical review. Dement Neuropsychol. 2019 June;13(2):133-143.

